# The emerging role of AI in enhancing intratumoral immunotherapy care

**DOI:** 10.18632/oncotarget.28643

**Published:** 2024-09-17

**Authors:** Abin Sajan, Abdallah Lamane, Asad Baig, Korentin Le Floch, Laurent Dercle

**Keywords:** interventional radiology, artificial intelligence, radiology, radiomics, intratumoral immunotherapy, precision Medicine

## Abstract

The emergence of immunotherapy (IO), and more recently intratumoral IO presents a novel approach to cancer treatment which can enhance immune responses while allowing combination therapy and reducing systemic adverse events. These techniques are intended to change the therapeutic paradigm of oncology care, and means that traditional assessment methods are inadequate, underlining the importance of adopting innovative approaches. Artificial intelligence (AI) with machine learning algorithms and radiomics are promising approaches, offering new insights into patient care by analyzing complex imaging data to identify biomarkers to refine diagnosis, guide interventions, predict treatment responses, and adapt therapeutic strategies. In this editorial, we explore how integrating these technologies could revolutionize personalized oncology. We discuss their potential to enhance the survival and quality of life of patients treated with intratumoral IO by improving treatment effectiveness and minimizing side effects, potentially reshaping practice guidelines. We also identify areas for future research and review clinical trials to confirm the efficacy of these promising approaches.

## INTRODUCTION

The emergence of immunotherapies (IO) has transformed cancer treatment. Assessing their effectiveness remains challenging due to atypical response and progression patterns. Traditional methods based on tumor size are inadequate for capturing unique dynamics of response, such as pseudoprogression, hyperprogression, and abscopal effect [1]. Intratumoral IO specifically plays a crucial role by directly modulating the tumor microenvironment to enhance immune response. In this editorial, we unravel the potential role of advances in artificial intelligence (AI) to enhance clinical care in patients treated with IO.

### Intratumoral IO

Intratumoral IO, involving direct injections of immunostimulatory agents into tumors, offers a promising alternative to systemic IO like checkpoint inhibitors by potentially transforming “cold” tumors into “hot” ones, enhancing immune response while minimizing systemic exposure and side effects. This method reduces the amount injected, mitigates costs, and allows for combination therapy without increased toxicity [2]. Despite its benefits, challenges remain such as the unpredictability of dominant cancer epitopes and less extensive long-term data compared to systemic therapies. Intratumoral approaches aim to enhance efficacy and patient tolerance by localizing treatment, minimizing adverse events, and providing a cost-effective treatment option.

### Emerging role of AI/radiomics in IO

The integration of AI and radiomics into IO offers significant advancements by analyzing complex imaging data to identify novel biomarkers and predict treatment responses. AI can allow for interpreting subtle changes in tumor characteristics that traditional metrics like size may not capture, thus enhancing the understanding of tumor behavior under IO [3]. However, these technologies face challenges such as complexity and potential overfitting, underspecification, a lack of standardized methods which affects reproducibility, and high data requirements that demand large, diverse datasets. Despite these hurdles, the field is evolving rapidly, but it requires methodological refinements and comprehensive validation before these approaches can be seamlessly incorporated into clinical practice.

For instance, pivotal studies using KEYNOTE-002 and KEYNOTE-006 have showcased the effectiveness of AI in analyzing CT images to predict overall survival in patients with advanced melanoma treated with systemic IO, achieving superior accuracy in survival estimates compared to traditional methods [4]. However, the application of AI for predicting outcomes in patients undergoing intratumoral IO remains an area of ongoing research, indicating a potential yet unexplored avenue for enhancing treatment assessment.

### Role of image-guided interventions in the age of precision medicine

**Figure 1:**
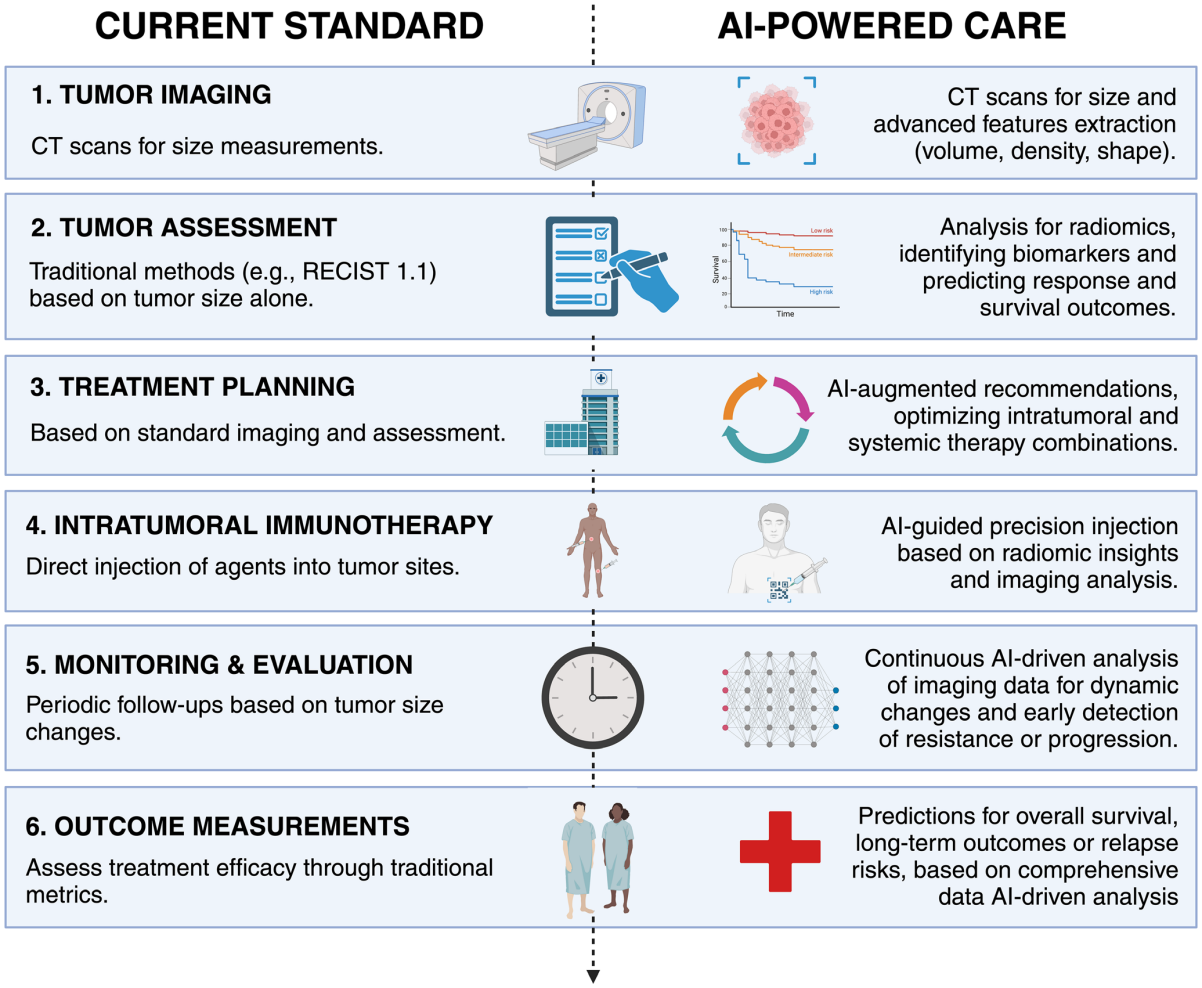
Transforming precision oncology: From conventional methods to AI-enhanced intratumoral immunotherapy.

AI can significantly enhance the care of patients treated with intratumoral IO by optimizing image-guided interventions. AI/radiomics offer advanced techniques that extract detailed information from standard imaging, such as CT scans, which traditional methods may overlook (Figure 1). These AI-driven techniques can predict subtle changes in the tumor environment, including the identification of key biomarkers, tissue composition, and variations in tumor heterogeneity over time. Specifically, AI can guide the precise delivery of intratumoral injections, targeting the most relevant tumor sites and adapting to the unique tumor landscape of each patient. This targeted approach not only maximizes the therapeutic efficacy by transforming “cold” tumors into “hot” ones that are more responsive to the immune system but also minimizes systemic toxicity and adverse effects compared to systemic therapies. However, the integration of AI in this field is still in its developmental stages, requiring further research to refine these technologies and validate their clinical benefits through rigorous trials. This ongoing evolution promises a move toward more personalized, effective, and less invasive cancer treatment modalities [5].

## CONCLUSIONS

The rapid evolution of cancer care, particularly with the advent of IO, presents new complexities that traditional diagnostic and treatment evaluation methods cannot adequately address. The integration of AI, especially through machine learning and radiomics, heralds a transformative approach in oncology. These technologies enable the extraction of complex data from imaging studies, leading to more precise diagnostics, better prediction of treatment responses, and tailored therapeutic strategies. As AI techniques mature and become integrated into clinical workflows, they promise to refine the way oncologists approach cancer treatment across various tumor types and treatment modalities. The potential to enhance patient outcomes and transform practice guidelines significantly is immense, particularly by improving the efficacy of treatments and reducing adverse effects. Ultimately, the sophisticated application of AI in IO could lead to improved survival rates and quality of life for cancer patients, setting a new standard in personalized medicine.
